# Silencing epileptic storms: targeting miRNA-lncRNA crosstalk in astrocytes and microglia to disarm neuroinflammatory triggers

**DOI:** 10.3389/fnmol.2025.1616804

**Published:** 2025-07-28

**Authors:** Jing Meng, Wen Luo, Nana Zhang, Mingxing Yu, Yuxuan He, Chunyan Chen, Haifeng Shu, Liang Yu

**Affiliations:** ^1^Department of Neurology, Sichuan Provincial People’s Hospital, School of Medicine, University of Electronic Science and Technology of China, Chengdu, Sichuan, China; ^2^Medical and Life Sciences, Chengdu University of Traditional Chinese Medicine, Chengdu, Sichuan, China; ^3^Department of Neurosurgery, The General Hospital of Western Theater Command, College of Medicine, Southwest Jiaotong University, Chengdu, China

**Keywords:** epilepsy, microRNAs, long noncoding RNAs, glial cell polarization, neuroinflammation, exosomes

## Abstract

Epilepsy is a chronic neurological disorder characterized by abnormal synchronous discharges of neurons in the brain. It affects approximately 70 million people worldwide, and approximately 30% of patients are resistant to existing antiepileptic drugs. Repeated seizures can lead to neuronal damage, glial cell activation and neuroinflammation, creating a vicious cycle of seizures, inflammation, and neuronal damage. Recent studies have shown that microRNAs play a key role in the pathological process of epilepsy by regulating the phenotype, inflammatory response and metabolic function of astrocytes and microglia. In addition, long noncoding RNAs, as upstream regulators of miRNAs, influence miRNA function by acting as competitive endogenous RNAs, further regulating glial cell activation and inflammatory responses. This paper is the first to systematically elucidate the synergistic role of miRNAs and lncRNAs in epilepsy through glial cell polarization, metabolic imbalance and exosome-mediated transcellular communication, providing a theoretical framework for the development of multitargeted intervention strategies.

## 1 Introduction

Epilepsy, a chronic neurological disorder characterized by recurrent seizures due to aberrant neuronal hyperexcitability, affects more than 70 million individuals globally, and approximately 30% of patients are resistant to currently available therapies (Espinosa et al., 2025). While neuronal dysfunction remains a central focus in the study of epilepsy, glial cells—astrocytes and microglia—have emerged as pivotal drivers of epileptogenesis, orchestrating neuroinflammation, synaptic remodelling, and metabolic imbalance (Hui Yin et al., 2013; McCormick and Contreras, 2001; Grillo, 2014). Activated astrocytes disrupt glutamate homeostasis due to impaired transporter function, leading to excitotoxic neuronal damage, whereas microglia are polarized toward the proinflammatory (M1) phenotype and release cytokines (e.g., IL-1β and TNF-*α*) that exacerbate blood–brain barrier leakage and synaptic hyperexcitability (Ghouli and Binder, 2025; Chen et al., 2017). This glia-centric “seizure-inflammation-neuronal injury” axis underscores the urgent need for multitargeted interventions to disrupt the cycle of pathological events (Scheid and Teich, 2007; Alsharafi et al., 2015; Hennebelle et al., 2014).

Within this axis, noncoding RNAs—microRNAs (miRNAs) and long noncoding RNAs (lncRNAs)—serve as master regulators of glial plasticity. miRNAs, small (~22 nt) posttranscriptional silencers (Martino et al., 2025), directly modulate glial gene expression. Despite increasing insights into the individual functions of miRNAs and lncRNAs, their synergistic regulation of glial crosstalk in epilepsy remains unknown. Previous studies have differentiated these mechanisms—focusing solely on miRNA-mediated neuronal excitability or lncRNA-driven blood–brain barrier (BBB) disruption—while neglecting the integrated glial network. This review is the first to delineate the cooperative roles of miRNAs and lncRNAs in glial-mediated epileptogenesis through three mechanisms: (1) Polarization control, i. e., bidirectional regulation of astrocyte A1/A2 and microglial M1/M2 polarization (e.g., miR-146a/Notch-1 vs. lncRNA SNHG5/NF-κB); (2) Metabolic rewiring, i. e., the modulation of glutamate uptake (miR-181c-5p/GLT-1) and oxidative stress (lncRNA UCA1/Nrf2) through miRNA–lncRNA networks; (3) Exosomal communication, i.e., propagation of inflammation through neuronal-glial communication via extracellular vesicles (EVs) carrying miR-155 or lncRNA ILF3-AS1.

By integrating the function of glia with that of noncoding RNAs, this work provides a transformative perspective for targeting the glial-RNA axis in drug-resistant epilepsy, bridging molecular discovery to therapeutic innovation.

## 2 miRNA-mediated regulation of astrocytic function

**Figure 1:**
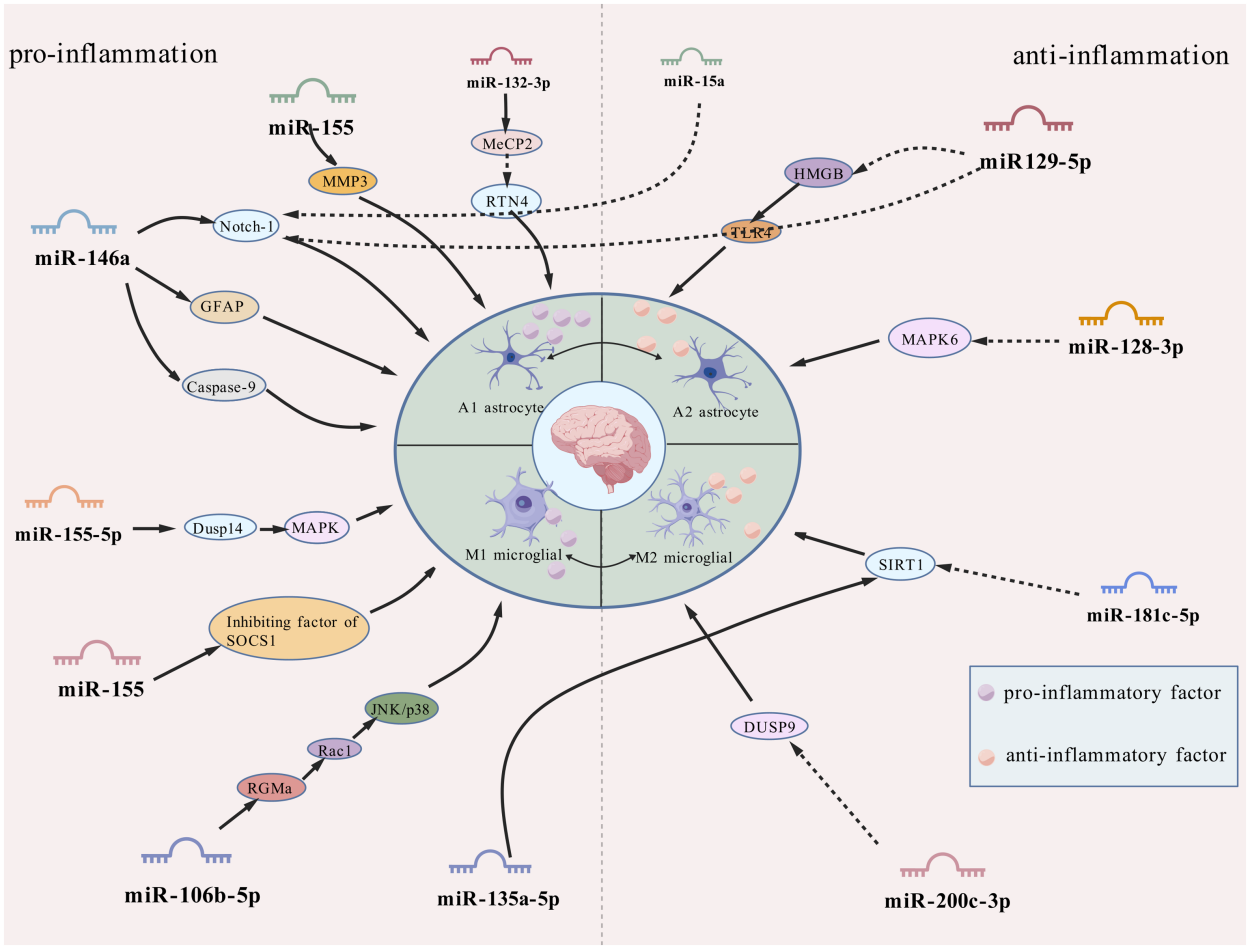
Mechanisms of lncRNA in glial cell polarization during epilepsy.

As the most numerous glial cell population in the central nervous system (CNS), astrocytes are widely distributed in normal neural tissues and play several key physiological regulatory roles. In the brain, these cells not only participate in the regulation of neurohomeostasis by maintaining neuronal structural integrity and promoting functional activities, but also play important roles in the regulation of ionic homeostasis, energy metabolism, synaptic network assembly and neurotransmitter transmission (Murphy-Royal et al., 2015; Eberhard et al., 2025). Notably, this class of glial cells with immunomodulatory properties has shown significant pathological relevance in several human diseases, and in particular their involvement in epileptic pathomechanisms by mediating the neuroimmune response has been confirmed by several studies (Shi et al., 2025). *In vitro* experimental data have shown that astrocytes in the activated state have significant inflammatory factor-secreting properties, a finding that is consistent with the abnormally high expression of pro-inflammatory mediators, such as interleukin-1β (IL-1β) and tumour necrosis factor-*α* (TNF-α), in experimental epilepsy models and epileptogenic human brain tissue (Zhou et al., 2018). Recent research has shown that astrocyte dysfunction is closely associated with the pathological onset and progression of epilepsy, suggesting a central regulatory role in the pathogenesis of the disease (Shen et al., 2016). Some researchers have suggested that regulating the polarisation status of astrocytes may be a novel intervention strategy for the treatment of refractory epilepsy, and this therapeutic concept may also be applicable to other brain injury disorders involving glial cell polarisation (Zhang et al., 2022). Abnormally activated astrocytes in pathological states have been shown to exacerbate neuroinflammatory responses through the sustained release of pro-inflammatory cytokines. Experimental evidence suggests that this hyperactivated state is an important driver of inflammation-mediated neuronal degeneration (Devinsky et al., 2013). Moreover, it has been demonstrated that inflammatory over activation of astrocytes can result in impaired glutamate transport, which, in turn, can trigger abnormal neuronal firing and ultimately induce epileptiform seizures (Sanz and Garcia-Gimeno, 2020). More information regarding the mechanisms by which miRNAs regulate astrocyte function is shown in Figure 1 and Table 1.

**Table 1:** Functional roles of miRNAs in glial cells.

miRNA	Levels	Targets	Levels	Effects on glial cells	Model	Epilepsy type	References
miR-132-3p	↑	MeCP2 RTN4	MeCP2↓ RTN4↑	A1 polarization↑	*In vivo*	SE	Zhang et al. (2022)
miR155	↑	MMP3	↑	A1 polarization↑	*In vivo*	TLE	Korotkov et al. (2018)
miR-146a	↓	Notch-1 GFAP Caspase-9	↓	A1 polarization↓	*In vivo*	TLE	Liu et al. (2017)
miR-15a	↑	GFAP	↓	A1 polarization↓	*In vivo*	–	Fan et al. (2020)
miR129-5p	↑	HMGB1/TLR4 Notch	↓	A1 polarization↓	*In vivo*	SE	Wan and Yang (2020)
miR-128-3p	↑	MAPK6	↓	A1 polarization↓	*In vitro*	–	Pang et al. (2022)
miR-181c-5p	↑	PKCδ/GLT-1	↓	Diminished glutamate uptake by astrocytes	*In vitro*	–	Ma et al. (2024)
miR-155-5p	↑	GLAST	↓	Diminished glutamate uptake by astrocytes	*In vivo*	–	Gao et al. (2017)
miR-22	↑	P2X7R	↓	Enhanced glutamate uptake by astrocytes	*In vivo*	MTLE-HS	Guerra Leal et al. (2022)
miR-155	↑	Inhibiting factor of SOCS1	↓	M1 polarization↑	*In vivo*	TBI	Aloi et al. (2023)
miR-200c-3p	↑	DUSP9	↓	M2 polarization↑	*In vivo*	–	Du et al. (2019)
miR-106b-5p	↑	RGMa-Rac1-JNK/p38 MAPK	↑	M1 polarization↑	*In vivo*	SE	Yu T. et al. (2022)
miR-135a-5p	↓	SIRT1	↓	Promotion of microglia apoptosis	*In vitro*	–	Wang et al. (2021)
miR-181c-5p	↑	SIRT1	↓	M1 polarization↓	*In vivo*	–	Kong et al. (2020)

### 2.1 Proinflammatory miRNAs drive astrocyte 1 polarization

**Figure 2:**
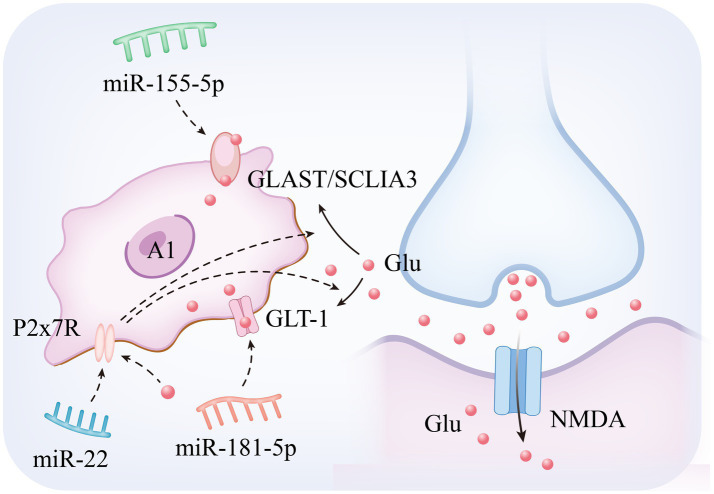
miRNA regulates imbalance of glutamate metabolism in astrocytes.

miR-132-3p: In a rat model of lithium-pilocarpine-induced sustained status epilepticus (SE), it was found that abnormally high expression of miR-132-3p drove the secretion of IL-1β and TNF-*α* from type A1 astrocytes, exacerbating neuronal damage and seizures by targeting gene methylated CpG-binding protein 2 (MeCP2) (down) and repressor reticulin 4 (RTN4) (up) (Wanet et al., 2012). Notably, targeted silencing of miR-132-3p (antagomiR-132) using specific inhibitors effectively inhibited the polarization process of type A1 astrocytes, seizure severity, and recurrence, highlighting therapeutic potential (Zhang et al., 2022; Figure 2).

miR-155: in the microenvironment surrounding human brain tissue lesions, miR-155 is mainly localized in the cytoplasm of activated astrocytes, and pathologically (Korotkov et al., 2020; Huang L. G. et al., 2018). It regulates the inflammatory activation status of astrocytes and occupies a central regulatory position in neuroinflammatory pathology (Ammothumkandy et al., 2025). On the one hand, it can reduce the intensity of the neuroinflammatory response by inhibiting the release of pro-inflammatory mediators from type A1 astrocytes, and on the other hand, it can maintain the physiological function of the BBB-ECM complex structure by down-regulating the expression of MMP3, which ultimately realizes a multidimensional intervention on epilepsy-related pathological damage (Korotkov et al., 2018; Toledo et al., 2025).

miR-146a: the study found that the overexpression of miR-146a is positively correlated with the severity of neuroinflammation (Perry et al., 2008; Saba et al., 2014). This neuroprotective mechanism involves coordinated regulation of downstream effectors: (1) Notch signaling modulation: The pivotal role of the pathway in neural stem cell maintenance and glial-neuronal lineage specification is disrupted through miR-146a silencing, which targets complementary binding sites within the 3′-untranslated regions (3’-UTRs) of Notch-1 mRNA. (2) Astrocytic activation control: Glial fibrillary acidic protein (GFAP), a hallmark of reactive astrogliosis, exhibits pathological overexpression in epileptic tissues that correlates with neuroinflammatory exacerbation (Venkatesh et al., 2013; Ahmadian et al., 2019; Hol and Pekny, 2015). miR-146a downregulation attenuates hippocampal gliosis by suppressing GFAP expression. (3) Apoptotic pathway regulation: Elevated caspase-9 activity in mesial TLE patients, positively associated with seizure frequency (Wetherington et al., 2008), is counteracted by miR-146a-mediated inhibition of caspase-9 signaling, reducing neuronal apoptosis. Silencing miR-146a attenuates gliosis and neuronal apoptosis, though specific inhibitors need development (Vega-García et al., 2021; Huang et al., 2019). Future investigations should prioritize high-precision miR-146a targeting approaches, coupled with cross-species validation and comprehensive toxicological profiling to advance translational potential.

### 2.2 Anti-inflammatory miRNAs attenuating astrocyte activation

miR-15a: it was found that miR-15 was significantly down-regulated in epilepsy and could serve as a potential biomarker for seizures (Ma, 2018; Cui et al., 2019). Mechanistically, miR-15a parallels the regulatory activity of miR-146a by directly binding complementary sequences within the 3′-untranslated region (3’-UTR) of GFAP mRNA, thereby suppressing posttranscriptional regulation and attenuating neurotoxic A1 astrocytic polarization. miR-15a/GFAP has great therapeutic potential as a key regulatory pathway in epileptic pathophysiology against seizures (Fan et al., 2020).

MiR-129-5p: immunohistochemical analyses demonstrate that exosome-derived miR-129-5p administration reverses SE-induced upregulation of glial activation markers while attenuating hippocampal IL-1β, IL-6, and TNF-*α* levels—effects mediated through HMGB1/TLR4 axis suppression and downstream inflammatory pathway inhibition (Liu et al., 2024; Wu et al., 2022). Upstream regulatory mechanisms involve the lncRNA NEAT1, which elevates IL-6/COX-2/TNF-α production via miR-129-5p sequestration and Notch pathway activation in epileptic networks (Wan and Yang, 2020). These findings collectively position miR-129-5p as a multimodal anti-inflammatory agent with therapeutic potential for SE, suggesting that pharmacological targeting of NEAT1 or disruption of NEAT1-miR-129-5p interactions could yield combined neuroprotective and anti-inflammatory benefits.

miR-128-3p: the mitogen-activated protein kinase (MAPK) cascade plays critical regulatory roles in inflammatory processes and other cellular functions (Kyriakis and Avruch, 2001). Clinical and experimental studies document consistent downregulation of miR-128-3p in both TLE patients and rodent models throughout disease progression (Alsharafi and Xiao, 2015). Mechanistic investigations reveal MAPK6 expression counteracts the neuroprotective effects exerted by miR-128-3p in this cellular model (Pang et al., 2022).

### 2.3 miRNA-mediated dysregulation of astrocytic glutamate homeostasis

Among the mechanisms regulating neurotransmitter homeostasis in epileptogenesis, astrocytes maintain the dynamic homeostasis of extracellular glutamate through their membrane-localized excitatory amino acid transporters (Glutamate Aspartate Transporter and Glutamate Transporter 1), and functional abnormalities of this system have been demonstrated to be an important pathological basis for hippocampal dysfunction and epileptogenesis (Hennebelle et al., 2014). Mechanistic analyses have shown that Glutamate Transporter 1 (GLT-1), as a core executive of the glutamate clearance system in the CNS, mainly undertakes the function of synaptic interstitial glutamate reuptake. Experimental studies have shown that specific inhibition of GLT-1 expression in astrocytes significantly elevates extracellular glutamate concentration, leading to a significant increase in seizure frequency and duration in epilepsy model animals, and restoration of glutamate transporter function through enhancement of GLT-1 stability has become an important research direction in antiepileptic therapy (Chotibut et al., 2014; Shibata et al., 1997; Norris et al., 2025). In pathological states, the expression and function of Glutamate Aspartate Transporter (GLAST) and GLT-1 are characterized by significant downregulation. This abnormality is closely related to the delivery mechanism of miRNAs within EVs secreted by pathogenic neurons: epilepsy-derived EVs deliver specific miRNAs to astrocytes via transcellular transport, which in turn inhibits the biosynthesis of glutamate transporter proteins and ultimately impairs the glutamate uptake capacity of glial cells (Seifert et al., 2006; Peghini et al., 1997; Wang F. et al., 2020; Ma et al., 2024). The resulting imbalance in glutamate homeostasis induces aberrant neuronal depolarization via NMDA receptor overactivation and drives synaptic plasticity remodeling (e.g., pathological alterations such as characteristic hippocampal mossy fiber outgrowths), which significantly reduces seizure threshold (Peterson and Binder, 2020; Peterson et al., 2021). These findings not only confirm the centrality of the astrocyte glutamate transport system (GLAST/GLT-1 axis) in epilepsy regulation, but also provide a theoretical framework for the development of novel therapeutic strategies for glial cell targeting (Shen et al., 2016). The specific mechanism is illustrated in Figure 2. Based on the above mechanisms, recent studies have been devoted to analyzing the key miRNAs targets that regulate glutamate metabolism in astrocytes, and the following are the research progresses with important translational value:

miR-181c-5p: in the multidimensional regulatory network of neurological diseases, miR-181c-5p is not only involved in the pathological process of Alzheimer’s disease (Manzano-Crespo et al., 2019), but also exhibits an important regulatory function in epileptogenesis and development. In recent years, it has been found that EVs act as key delivery vehicles for miRNAs (Yu D. et al., 2022), mediating intercellular communication to achieve gene expression regulation and biological function remodeling (Yang et al., 2019). A typical paradigm can be seen in the neuron–glial cell interaction system: neuron-derived EVs, after internalized and uptaken by astrocytes, significantly enhance glutamate transport efficacy, which in turn achieves negative feedback regulation of neuronal electrophysiological activities (Men et al., 2019). Of particular interest, EVs released from epilepsy-derived neurons can specifically act on the protein kinase Cδ (PKCδ) /GLT-1 signaling axis in astrocytes by transporting miR-181c-5p, leading to impaired glial glutamate clearance and ultimately significantly elevating epilepsy susceptibility (Ma et al., 2024). This finding not only reveals a novel molecular mechanism of neuron–glia interaction in epileptic processes, but also lays a theoretical foundation for the development of therapeutic strategies targeting the contents of EVs, such as miRNA inhibitors or GLT-1 agonists.

miR-155-5p: MiR-155-5p (i.e., miR-155) exhibits pleiotropic regulatory features, and its function involves multiple pathological dimensions such as diffuse large B-cell lymphoma, imbalance of immune homeostasis (Ji et al., 2015), and tumor microenvironment regulation (Gasparini et al., 2014; Fang et al., 2024). Mechanistic studies have shown that activator protein-1 (AP-1), as a transcriptional regulatory complex composed of members of the JUN/FOS family, plays a central regulatory role in biological processes such as inflammatory response, cell proliferation, and programmed death by forming heterodimeric complexes that bind to specific DNA sequences (Karin et al., 1997). Of particular note, a pentylenetetrazol (PTZ)-induced epilepsy model demonstrated a significant antiepileptic effect, and its mechanism of action involves a targeted intervention in the AP-1/miR-155-5p/GLAST signaling cascade network: the compound reduces the downstream miR-155-5p expression level by inhibiting the AP-1 transcriptional activity, and at the same time reverses the miRNA inhibitory effect on the glutamate transporter GLAST in astrocytes. This dual regulatory action ultimately effectively corrected PTZ-induced disorders of glutamate metabolism and epileptiform seizures by restoring the efficacy of extracellular glutamate clearance, revealing the translational potential of the AP-1/miR-155-5p/GLAST pathway as a novel target for epilepsy therapy (Gao et al., 2017).

miR-22: miR-22 exhibits neuroprotective and neuromodulatory roles across epilepsy subtypes. Preclinical studies reveal reduced circulating miR-22 levels in refractory epilepsy models (Jimenez-Mateos et al., 2015a), consistent with its established antiapoptotic function in traumatic brain injury paradigms (Ma et al., 2016). Mechanistically, miR-22 modulates neuronal hyperexcitability, neuroinflammatory responses, and maladaptive neurogenesis—processes driven by ATP-mediated P2X7 receptor (P2X7R) activation (Jimenez-Mateos et al., 2015a). In drug-resistant epilepsy, neocortical P2X7R overexpression disrupts glutamate-GABA homeostasis by (1) reducing astrocytic glutamate uptake and (2) suppressing GABA synthesis, thereby enhancing GABAergic signaling and destabilizing excitatory-inhibitory balance (Barros-Barbosa et al., 2016). This miR-22/P2X7R regulatory axis, potentially mediated through posttranslational modification mechanisms, emerges as a therapeutic target for refractory MTLE-HS. Pharmacological intervention targeting this interaction may advance development of next-generation antiepileptic therapies (Guerra Leal et al., 2022; Samões et al., 2024).

## 3 miRNAs regulate microglial phenotypic switching in epileptic neuroinflammation

In the regulatory network of neuroinflammation, microglia act as core effector cells, and their phenotypic polarization process is precisely regulated by miRNAs and long chain non-coding RNAs (lncRNAs) (Terrone et al., 2017; Peng et al., 2019). These immunoreactive cells not only possess the function of secreting multiple cytokines/chemokines, but also sense the dynamic changes of inflammatory signaling molecules within the CNS (Hanisch, 2002). After brain injury of epileptic origin, these cells become a major source of pro-inflammatory cytokine secretion, and the inflammatory mediators they release not only enhance the neuronal excitability threshold, but also directly contribute to the development of abnormal discharge activity, which has been recognized as a key driver of epilepsy formation (Colonna and Butovsky, 2017). Microglia in the CNS are highly plastic and are capable of polarizing toward different phenotypes such as classically activated (M1) type and alternatively activated (M2) type (Ma et al., 2017). Among them, the M1 type exacerbates the neuroinflammatory process by releasing pro-inflammatory mediators (Kalkman and Feuerbach, 2016), whereas the M2 type secretes immunosuppressive factors, such as TGF-*β* and IL-10, and is involved in the regulation of glioma cell proliferation (Lan et al., 2017; Wesolowska et al., 2008). Studies have shown that in the hypoxic microenvironment of glioblastoma (GBM), tumor cells can induce tumor-associated macrophages to polarize toward the M2 type through the secretion of paracrine factors such as periostin (POSTN) and exosomes (Guo et al., 2016). In terms of miRNA regulatory mechanisms, miR-181c-5p effectively inhibited microglia overactivation in a sepsis model by targeting high mobility group protein B1 (HMGB1), while decreasing the levels of inflammatory factors, such as TNF-*α* and IL-1β, and decreasing hippocampal neuronal apoptosis (Li et al., 2021). These findings reveal the multidimensional regulatory properties of miRNAs in epilepsy pathology, and their specific mechanisms of action and potential as therapeutic targets will be systematically described below.

### 3.1 Proinflammatory miRNA axes amplifying microglial M1 polarization

miR-155: within epileptic neuroinflammatory networks, miR-155 emerges as a dual-functional regulator coordinating both proinflammatory responses and programmed cell death, serving as a pivotal molecular nexus in epileptogenic processes. Beyond its astrocytic overexpression (Korotkov et al., 2020), this miRNA amplifies cerebral inflammatory cascades through glial activation modulation, potentially contributing to secondary injury mechanisms post-traumatic brain injury (TBI). Mechanistic investigations reveal microglial miR-155 induces M1 polarization by suppressing Suppressor of Cytokine Signaling 1 (SOCS1) expression, thereby exacerbating neuroinflammation (Sun et al., 2018a). Paradoxically, *in vitro* microglia-specific miR-155 knockdown triggers electrophysiological dysregulation, manifested by accelerated epileptogenesis onset, prolonged seizure duration, and elevated mortality—evidence of its bidirectional regulatory complexity (Aloi et al., 2023).

Notably, miR-155-5p (mature miR-155 isoform) demonstrates significant upregulation in both epileptic animal models and human medial TLE hippocampal tissues (Huang L. G. et al., 2018). Dusp14, as a MAPK pathway negative regulator, its dysfunction intensifies neurological damage (Kumar et al., 2021; Shi et al., 2022). miR-155-5p also drives neuronal inflammation and apoptosis through inhibition of Dusp14-mediated MAPK hyperphosphorylation while aggravating seizure severity and recurrence (Fang et al., 2024). Paradoxically, in vitro microglia-specific miR-155 knockdown triggers electrophysiological dysregulation, manifested by accelerated epileptogenesis onset, prolonged seizure duration, and elevated mortality—evidence of its bidirectional regulatory complexity (Aloi et al., 2023).

miR-200c-3p: in the study of cross-disease regulatory mechanisms between glioma and epilepsy, miR-200c-3p, as an important member of the miR-200 family (Cheng et al., 2016), exhibits unique double-edged sword-like regulatory properties: (1) During the pathological process of GBM, the molecule drives the remodeling of the tumor microenvironment through the exosome-mediated neuron–microglia communication network. The specific mechanism is as follows: neuron-derived exosomes deliver miR-200c-3p to microglia, leading to a reduction in the level of mRNA methylation modification by inhibiting the expression of the zinc finger protein ZC3H13 (a key regulator of m6A methylation), which in turn down-regulates the expression of Dual-Specificity Phosphatase 9 (DUSP9). loss-of-function of DUSP9 activates the ERK signaling pathway that induces microglia polarization toward a pro-tumorigenic M2 phenotype, ultimately accelerating the malignant progression of GBM (Guo et al., 2024). (2) In epilepsy models, inhibition of miR-200c-3p expression exerts neuroprotective effects through a triple protective mechanism: (i) activation of the RECK/AKT signaling axis, up-regulation of the expression of cysteine-rich RECK proteins (inhibitors of matrix metalloproteinases), inhibition of the AKT phosphorylation cascade, and attenuate hippocampal neuronal damage (Alexius-Lindgren et al., 2014). (ii) regulate the activation status of glial cells, and inhibit the process of reactive gliosis by decreasing the expression level of GFAP, a marker of astrocyte activation; (iii) remodel the microenvironment of neuroinflammation, and significantly reduce the release of pro-inflammatory factors (Du et al., 2019).

miR-106b-5p: Clinical assay data show that miR-106b-5p is characterized by significantly high expression in the peripheral blood of epilepsy patients, and its pathological mechanism of action involves transcriptional repression of rejection guidance molecule A (RGMa), which in turn activates the RGMa-Rac1-JNK/p38 MAPK signaling cascade network (Li et al., 2017; Wang et al., 2015; Yu et al., 2021). This molecular cascade response drives microglia polarization toward a pro-inflammatory M1 phenotype, which induces a massive release of inflammatory mediators such as IL-1β and IL-6, ultimately leading to deterioration of the neuroinflammatory microenvironment and neuronal degeneration. Experimental studies revealed that specific inhibition of miR-106b-5p could remodel the microglial M1/M2 polarization balance, providing new ideas for the development of precision antiepileptic therapies based on glial cell phenotype modulation (Yu T. et al., 2022).

### 3.2 Anti-inflammatory miRNAs promoting M2 polarization

miR-22: miR-22 plays a key role in epilepsy pathology through the multidimensional regulation of inflammatory and metabolic functions in glial cells (Beamer et al., 2018). First, in astrocytes, miR-22 reduces the risk of seizures triggered by neuronal hyper excitability by regulating the expression of genes related to glutamate metabolism, increasing glutamate uptake and decreasing aberrant glutamate release, thereby maintaining the synaptic excitatory/inhibitory balance. Second, P2X7 is a receptor expressed by microglia, which can lead to the release of proinflammatory cytokines Il-1β and TNF-*α* and the production of reactive oxygen species (Monif et al., 2009; Choi et al., 2012). miR-22 negatively regulates P2X7 receptor expression by directly binding to the 3’-UTR of the P2X7 receptor and inhibiting its translation, thereby suppressing the inflammatory response (Guerra Leal et al., 2022). This dual regulatory mechanism (interfering with astrocyte metabolic abnormalities and inhibiting microglial inflammatory activation) provides a theoretical basis for the development and potential clinical translational value of novel antiepileptic therapies targeting the miR-22/P2X7 axis, particularly for patients with drug-resistant epilepsy.

miR-181a-5p: SIRT1 (sirtuin-1) is a protein deacetylase that regulates gene expression by catalysing the deacetylation of histone proteins. NAD + is required for the deacetylase activity of SIRT1, which is directly linked to CNS disorders. SIRT1 deficiency in microglia has been associated with cognitive decline during neurodegeneration (Cho et al., 2015). Increased hippocampal expression of miR-181a-5p was found in an immature rat model of lithium/pilocarpine-induced epilepsy. Inhibition of miR-181a-5p suppresses astrocyte and microglial activation by upregulating SIRT1, which plays a role in suppressing seizures and ameliorating cognitive decline in TLE patients (Kong et al., 2020).

miR-135a-5p: miR-135a-5p was found to induce apoptosis not only in glioma, ovarian cancer and cardiomyocytes but also in glial cells in epilepsy. In an *in vitro* model of epilepsy induced by kainic acid (KA), miR-135a-5p expression is significantly upregulated, and an miR-135a-5p inhibitor effectively increases BV2 cell proliferation and inhibits apoptosis. Moreover, miR-135a-5p may also be involved in epilepsy-induced apoptosis through the SIRT1-related signalling pathway. siRNA-SIRT1 effectively inhibits the proliferation of BV2 microglia and promotes microglial apoptosis (Wang et al., 2021).

## 4 LncRNA-miRNA crosstalk in glial pathophysiology

**Figure 3:**
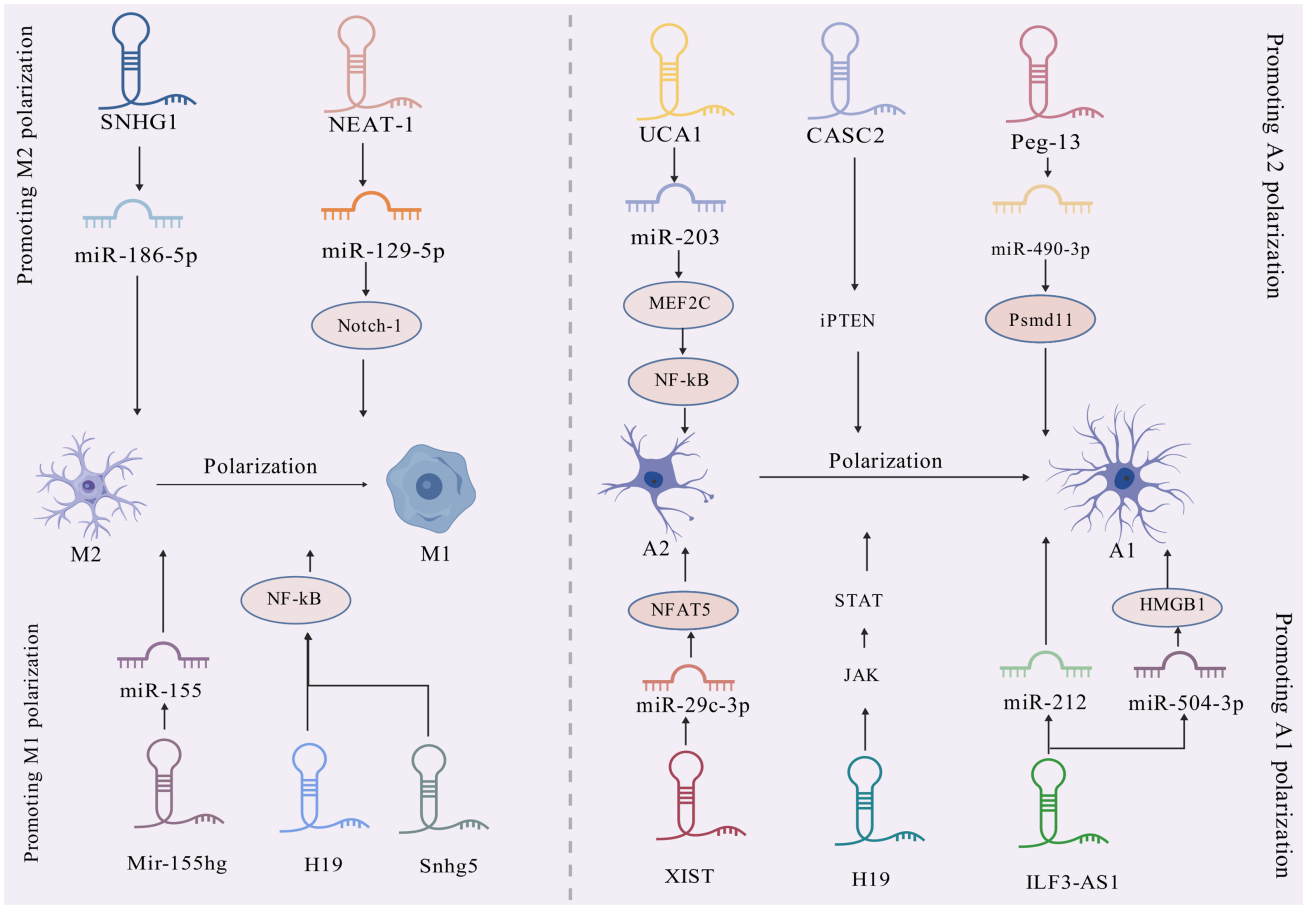
Mechanisms of lncRNA–miRNA crosstalk during glial cell polarization in epilepsy.

**Figure 4:**
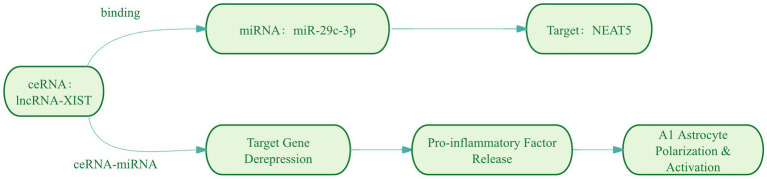
ceRNA serves as a “molecular switch” regulating cellular functions. LncRNA XIST functions as a ceRNA by binding to miR-29c-3p, leading to its functional sequestration. This derepresses miR-29c-3p target genes (e.g., NFAT5), ultimately promoting neurotoxic A1 astrocytic polarization in epilepsy.

Mechanistically, long noncoding RNAs (lncRNAs) serve as competitive endogenous RNAs (ceRNAs) by sequestering shared miRNAs that would otherwise target mRNAs, thereby influencing disease pathogenesis through posttranscriptional regulation (Tan and Marques, 2016). Within the CNS, microglia and astrocytes demonstrate functional plasticity through polarized activation states: The pro-inflammatory M1/A1 phenotypes drive neurotoxicity via inflammatory mediator release and oxidative damage, while the M2/A2 phenotypes exhibit neuroprotective capacities through anti-inflammatory actions and synaptic maintenance (Kwon and Koh, 2020). Emerging research identifies lncRNAs as upstream epigenetic regulators of miRNAs, positioning them as potential diagnostic biomarkers and therapeutic targets for neurological disorders such as Alzheimer’s disease, ischemic cerebrovascular events, and demyelinating conditions (Khan et al., 2022). Despite these advances, the precise regulatory networks through which lncRNAs govern neuroinflammatory processes via glial cell modulation in epilepsy remain poorly characterized. There exists a critical need for comprehensive analysis of lncRNA-mediated regulatory circuits during epileptogenesis, particularly those involving glial activation and subsequent inflammatory pathway dysregulation. Current understanding of lncRNA-mediated control over microglial and astrocytic functionality is summarized in Table 2 and Figure 3, and how lncRNAs influence miRNAs is summarized in Figure 4, revealing promising avenues for future mechanistic investigations.

**Table 2:** Functional roles of lncRNAs in glial cells.

lncRNA	Levels	Targets	Levels	The effects on glial cells	Models	Epilepsy type	References
SNHG1	↑	miR-186–5p	↓	M1 polarization↓ M2 polarization↑	*In vitro*	Ischaemic epilepsy	Bao et al. (2024)
Mir155hg	↑	Mir-155 NF-κB	↑	Inhibition of microglial phagocytic activity/ M1 polarization↑M2 polarization↓	*In vivo*	CSE	Wang et al. (2024) and Fang et al. (2024)
H19	↓	NF-κB	↓	M1 polarization↓	–	CSE	Xie et al. (2022)
XIST	↓	miR-29c-3p -NFAT5	↑	A1 polarization↓	In vivo	–	Zhang et al. (2021)
Peg13	↑	miR-490-3p--Psmd11	↑	A1 polarization↓	In vivo	–	Feng et al. (2020)
H19	↑	JAK/STAT	↑	A1 polarization↑	*In vitro*	TLE	Han et al. (2017) and Han et al. (2018b)
ILF3-AS1	↑	miR-212--MMP3/9	miR-212↓ MMP3/9↑	A1 polarization↑	*In vivo*	TLE	Cai et al. (2020)
UCA1	↑	miR-203/MEF2C/NF-κB	↓	A1 polarization↓	*In vitro*	–	Yu et al. (2020)
CASC2	↑	PTEN	↑	Astrocytes	*In vitro*	–	Zhu et al. (2020)
NEAT1	↑	miR-129-5p --notch	miR-129-5p ↓ notch↑	M1 polarization↑	*In vivo*	TLE	Wan and Yang (2020)
ILF3-AS1	↓	miR-504-3p/HMGB1	miR-504-3p↑ HMGB1 ↓	A1 polarization↓	*In vitro*	TLE	Gao et al. (2024)

LncRNA X-inactive-specific transcript (XIST): The X-inactive specific transcript (XIST), a long noncoding RNA encoded on the X chromosome, has emerged as a key epigenetic regulator of inflammatory pathways across multiple disease states (Zhang Y. et al., 2019). Mechanistic studies reveal XIST functionally sequesters miR-29c-3p, a miRNA notably downregulated in epileptic rat models and demonstrating an inverse correlation with XIST levels (Zhang et al., 2021). Functional experiments demonstrate that miR-29c-3p overexpression suppresses proinflammatory cytokine secretion in BV2 microglial cells, effectively counteracting lipopolysaccharide (LPS)-induced inflammatory cascades (Wang R. et al., 2020). Of particular therapeutic interest, this XIST/miR-29c-3p axis has been implicated in controlling astrocytic polarization toward the neurotoxic A1 phenotype, positioning these molecules as promising therapeutic candidates for epilepsy intervention (Zhang et al., 2021; Table 3).

**Table 3:** Abbreviations and corresponding full names in the text.

Abbreviation	Full name
AP-1	Activator protein-1
Arg-1	Arginase-1
BBB	Blood–brain barrier
ceRNA	Competing endogenous RNA
CSE	Convulsive status epilepticus
DUSP9	Dual-specificity phosphatase 9
EVs	Extracellular vesicles
GFAP	Glial fibrillary acidic protein
GLAST	Glutamate aspartate transporter
GLT-1	Glutamate transporter 1
HMGB1	High-mobility group box 1
IFN-γ	Interferon-gamma
IL-1β	Interleukin-1 beta
IL-6	Interleukin-6
JAK/STAT	Janus kinase/signal transducer and activator of transcription
LPS	Lipopolysaccharide
MAPK	Mitogen-activated protein kinase
MMP	Matrix metalloproteinase
MTLE-HS	Mesial temporal lobe epilepsy with hippocampal sclerosis
NF-κB	Nuclear factor kappa B
NLRP3	NOD-like receptor pyrin domain containing 3
Nrf2	Nuclear factor erythroid 2-related factor 2
PKCδ	Protein kinase C delta
PTZ	Pentylenetetrazol
SE	Status epilepticus
SOCS1	Suppressor of cytokine signaling 1
TLR4	Toll-like receptor 4
TNF-α	Tumor necrosis factor-alpha
TLE	Temporal lobe epilepsy
TSC	Tuberous sclerosis complex

LncRNA Peg13: The 26S proteasome non-ATPase regulatory subunit 11 (Psmd11) serves as a pivotal coordinator of cellular stress adaptation and inflammatory modulation (Wang et al., 2018; Wei et al., 2019). Experimental evidence from rodent epilepsy models reveals that hippocampal Psmd11 expression undergoes coordinated regulation through lncRNA Peg13-mediated molecular interactions. Functioning as a competitive endogenous RNA, Peg13 binds and sequesters miR-490-3p, thereby alleviating its inhibitory effect on Psmd11 transcripts. This post-transcriptional derepression elevates Psmd11 levels, which in turn: (1) Inhibits Wnt/*β*-catenin signaling-mediated A1 astrocyte polarization. (2) Attenuates neuroinflammatory cascades.

The resultant suppression of epileptogenic processes highlights the Peg13/miR-490-3p/Psmd11 axis as both a disease-promoting network and a viable therapeutic target for seizure disorders (Hodges and Lugo, 2018). Pharmacological validation studies further confirm its dual role in epilepsy pathophysiology and treatment development (Feng et al., 2020; Wang et al., 2018).

LncRNA ILF3-AS1: Matrix metalloproteinases (MMPs) constitute pivotal mediators of epileptogenic processes through their proteolytic regulation of neurovascular integrity. Pathological upregulation of MMP activity post-injury drives epileptogenesis via tripartite mechanisms: (1) extracellular matrix degradation, (2) BBB compromise, and (3) neuroimmune activation, as evidenced by elevated MMP2/3/9 levels in both TLE patients and experimental models (Dubey et al., 2017; Korotkov et al., 2018; Rempe et al., 2018). This mechanistic understanding positions MMP inhibitors as promising disease-modifying therapies for refractory epilepsy (Dubey et al., 2017).

Emerging evidence implicates the long noncoding RNA ILF3-AS1 as a master regulator of MMP-mediated epileptogenic processes in TLE pathogenesis. Multi-omics analyses demonstrate consistent ILF3-AS1 upregulation coupled with miR-212 downregulation in hippocampal tissues and serum samples from TLE patients versus controls (Cai et al., 2020). Functional studies reveal ILF3-AS1 exacerbates neuroinflammation and matrix remodeling through dual epigenetic mechanisms: (1) Sponging miR-212 to derepress MMP3/9 expression; (2) Sequestering miR-504-3p to amplify HMGB1-mediated oxidative stress.

These synergistic effects promote neuronal hyperexcitability in both *in vitro* epileptiform models (magnesium-deprived hippocampal neurons) and *in vivo* TLE paradigms (Gao et al., 2024; Cai et al., 2020).

LncRNA UCA1: the long noncoding RNA UCA1 demonstrates antiepileptic effects through multimodal suppression of astrocyte activation and neuroinflammation. Its protective mechanisms involve three primary pathways: (1) miR-203/MEF2C/NF-κB Axis: Myocyte enhancer factor 2c (MEF2C), a MADS/MEF2 family transcription factor critical for epilepsy regulation, modulates both neuronal excitability/synaptic plasticity and neuroinflammatory processes via inflammatory mediators and signaling networks (Xu et al., 2015). UCA1 forms a regulatory circuit by suppressing miR-203 (which directly inhibits MEF2C) (Luo et al., 2014; Gong et al., 2018), thereby enhancing MEF2C expression. This upregulation attenuates NF-κB pathway activity, limiting A1 astrocyte activation and subsequent release of proconvulsive factors like IL-1β that promote neuronal hyperexcitability (Bellot-Saez et al., 2017; Yu et al., 2020). (2) miR-495/Nrf2 Pathway: UCA1 mitigates seizure-associated neuronal apoptosis and cerebral damage via modulation of the miR-495/Nrf2 signaling axis (Geng et al., 2018). (3) JAK/STAT3 Signaling: Through inhibition of JAK/STAT3 activation, UCA1 downregulates astrocytic glutamate transporters (e.g., GLAST), reducing glial hyperactivation and neuroinflammatory cascades (Raymond et al., 2011; Wang H. et al., 2020).

Collectively, UCA1 counteracts epileptogenesis by targeting these interconnected pathways to suppress A1 astrocytosis, neuronal death, and neuroinflammation. These insights position UCA1 as a promising therapeutic target and underscore the potential of lncRNA-based strategies for epilepsy intervention.

miR-155hg: the long noncoding RNA miR-155hg serves as the precursor of miR-155, with its second exon directly encoding this miRNA. Both miR-155hg and miR-155 are critically involved in CNS disorders (Korotkov et al., 2020). miR-155hg is predominantly expressed in macrophages and microglia, where it plays a central role in maintaining brain homeostasis and modulating neuroinflammation. During convulsive status epilepticus (CSE), microglial phagocytic activity is essential for responding to neuronal injury and preserving brain homeostasis. Studies demonstrate that miR-155hg inhibits microglial phagocytic function, thereby delaying the clearance of damaged neurons and exacerbating hippocampal neuronal injury (Wang et al., 2024). Suppression of miR-155hg expression enhances microglial phagocytic activity, promotes neuronal repair, and reduces neuroinflammatory responses through regulation of miR-155 signaling, suggesting therapeutic potential for mitigating CSE progression (Wang et al., 2024).

LncRNA H19: functioning through immunoinflammatory and neuronal injury-associated pathways, H19 drives neuroglial hyperactivation via NF-κB-mediated inflammatory cascades (Han et al., 2017; Han et al., 2018b). In GBM tissues, this lncRNA promotes malignant phenotypes including glioma cell proliferation, migration, and invasion (Jiang et al., 2016). Within epileptic contexts, H19 exacerbates disease progression by dual mechanisms: (1) activating the JAK/STAT pathway to induce astrocytic GFAP expression and A1 polarization (Xu et al., 2011), and (2) enhancing microglial M1 polarization (evidenced by elevated CD86/iNOS) and astrocyte activation, which collectively amplify proinflammatory cytokine release (e.g., IL-1β, TNF-*α*). This creates a self-reinforcing cycle of gliosis, neuroinflammation, and hippocampal neuronal apoptosis, ultimately worsening seizure pathology (Han et al., 2018a; Vezzani and Friedman, 2011).

Experimental validation demonstrates that H19 knockdown suppresses NF-κB phosphorylation/nuclear translocation, reduces M1 marker expression, decreases neuronal apoptosis, and mitigates CSE-induced brain damage (Limanaqi et al., 2019). These findings establish H19 as a central regulator of TLE pathogenesis through glial phenotype modulation and NF-κB-dependent inflammation. Therapeutic targeting of H19 or its downstream effectors may disrupt this pathogenic network by restoring anti-inflammatory microenvironments and preserving neuronal integrity.

LncRNA Snhg5: the long noncoding RNA Snhg5, whose expression is dysregulated in inflammatory disorders, contributes to epileptogenesis through neuroinflammatory modulation (Shen et al., 2020). *In vitro* studies confirm that lipopolysaccharide (LPS) activates NF-κB signaling via p65 phosphorylation and nuclear translocation, facilitating proinflammatory gene transcription—consistent with established mechanisms (Sun et al., 2018b; Davari et al., 2020). Critically, Snhg5 silencing attenuates LPS-induced NF-κB pathway activation and exerts anticonvulsant effects by reprogramming microglial polarization. This dual modulation involves: (1) reducing proinflammatory M1 polarization (marked by CD86/iNOS downregulation) and associated cytokine release (IL-1β, TNF-*α*). (2) Enhancing anti-inflammatory M2 polarization (indicated by Arg-1/CD206 upregulation) and protective factor expression (e.g., IL-10). These shifts remodel the neuroinflammatory milieu, alleviating epilepsy-associated neuronal injury (Wang et al., 2022). Collectively, Snhg5 emerges as a pivotal regulator of microglial M1/M2 equilibrium via NF-κB signaling, highlighting lncRNA-targeted strategies to mitigate glia-driven neuroinflammation.

LncRNA NEAT1: the long noncoding RNA NEAT1, a critical regulator of paraspeckle formation in mammalian nuclei, plays a multifaceted role in glial-mediated neuroinflammation and neurological disorders through its functional dysregulation (Katsel et al., 2019). In oligodendroglial cells, NEAT1 maintains neural homeostasis by orchestrating myelin-related gene expression, with its aberrant activity linked to schizophrenia pathogenesis (Katsel et al., 2019). Within epileptic contexts, NEAT1 exacerbates neuroinflammatory responses and glial activation via two principal mechanisms: (1) Inflammasome activation: NEAT1 enhances NLRP3/NLRC4 inflammasome activity, triggering pyroptotic cell death and proinflammatory mediator release (IL-6, COX-2, TNF-α), which collectively induce oxidative stress and mitochondrial dysfunction (Zhang P. et al., 2019); (2) microglial polarization: by sequestering miR-129-5p to derepress Notch signaling, NEAT1 drives microglial M1 polarization, amplifying IL-1β secretion and aggravating seizure-associated neuroinflammation and neuronal injury (Wan and Yang, 2020). These coordinated actions position NEAT1 as a promising therapeutic target for epilepsy, given its dual regulatory capacity over glial functions (myelination regulation, phenotypic polarization) and inflammatory network modulation.

LncRNA SNHG1: small nucleolar RNA host gene 1 (SNHG1), a chromosome 11-derived lncRNA critical for 18S rRNA processing (Huang L. et al., 2018), demonstrates neuroprotective properties in cerebral ischemic pathologies (Zhang et al., 2018). Mechanistically, SNHG1 overexpression modulates the transcription factor YY1 (implicated in acute ischemic hippocampal injury) (Wan et al., 2019) through miR-186-5p sequestration, achieving dual immunoregulatory outcomes: (1) Suppression of proinflammatory mediators (TNF-α, IL-1β). (2) Enhancement of anti-inflammatory IL-10 production via microglial phenotype regulation. These effects collectively mitigate hypoxia-induced neuronal damage while preserving M2 microglial function. Notably, nanoengineered delivery systems for SNHG1 mimics present a novel therapeutic strategy for ischemia-related epilepsy (Zhang et al., 2018).

The emerging evidence collectively underscores that lncRNAs orchestrate epileptogenic processes through convergent mechanisms: (1) ceRNA-mediated miRNA sequestration (e.g., XIST/miR-29c-3p, Peg13/miR-490-3p, ILF3-AS1/miR-212), which derepresses downstream targets to drive glial polarization; (2) inflammatory pathway amplification via NF-κB (H19, Snhg5), JAK/STAT (H19), and inflammasome activation (NEAT1); and (3) dual regulation of neuronal excitability and neurovascular integrity through MMPs (ILF3-AS1), glutamate transporters (UCA1), and oxidative stress responses (UCA1/miR-495/Nrf2). Crucially, these lncRNAs form interconnected axes—such as NEAT1/miR-129-5p/Notch in microglial M1 polarization and H19/NF-κB in astrocytic A1 conversion—that create self-reinforcing cycles of gliosis and neuroinflammation. Their consistent dysregulation in epileptic tissues (e.g., ILF3-AS1↑ in TLE hippocampi, miR-155hg↑ in CSE) positions them not only as biomarkers but as master regulators of the “glial-RNA axis.” Therapeutic targeting of these nodes—via antisense oligonucleotides against H19/NEAT1 or engineered exosomes delivering UCA1/SNHG1 mimics—represents a promising paradigm for multitargeted intervention in drug-resistant epilepsy.

## 5 Exosomal noncoding RNAs: biomarker discovery and therapeutic engineering in epilepsy

Neuroinflammation constitutes a pivotal pathological mechanism in epilepsy, where inflammatory cascades frequently drive disease progression and seizure recurrence. Targeting neuroinflammatory pathways represents a promising disease-modifying strategy, supported by (1) demonstrated efficacy of anti-inflammatory drugs in drug-resistant epilepsy (Vezzani et al., 2019), (2) preclinical evidence showing reduced seizure frequency and enhanced neuroprotection via anti-inflammatory interventions (Terrone et al., 2020), and (3) regulatory potential of noncoding RNAs (miRNAs/lncRNAs) in inflammatory modulation. However, therapeutic application of free miRNAs faces challenges including (1) rapid degradation by serum nucleases and lysosomal pathways, (2) limited BBB permeability, and (3) poor target specificity.

Exosomes (30–100 nm vesicles) address these limitations through (1) endogenous phospholipid bilayer protection against enzymatic degradation, (2) bidirectional BBB penetrability without structural modification, and (3) natural tropism via membrane-bound ligands (Shyam et al., 2025). Clinically, exosomal biomarkers exhibit diagnostic value through (1) elevated miR-134 levels in TLE serum, exacerbating neuronal hyperexcitability via LIMK1 inhibition (Jimenez-Mateos et al., 2012), (2) upregulated miR-146a/miR-155 correlating with neuroinflammation severity and Electroencephalogram abnormalities (Omran et al., 2012; Liu et al., 2022), and (3) downregulated forebrain miR-346/miR-331-3p levels (Gitaí et al., 2020). Therapeutically, exosome advantages over synthetic nanoparticles include (1) reduced immunogenicity and macrophage clearance, (2) prolonged circulatory half-life, and (3) versatile cargo-loading capacity for proteins/nucleic acids (Xian et al., 2019; Ou et al., 2020). These dual diagnostic-therapeutic properties position exosomes as transformative tools for precision epilepsy management.

Inhibiting the expression of pathogenic miRNAs is a promising therapeutic strategy for example. For example, the delivery of miR-134 antisense oligonucleotides (antagomirs) via exosomes significantly reduces spontaneous seizures and protects hippocampal neurons from damage in an epilepsy mouse model (Jimenez-Mateos et al., 2015b). Similarly, exosome-mediated delivery of inhibitors of miR-155 inhibits microglial activation and attenuates epilepsy-associated inflammation (Liu et al., 2022). In addition, measuring miR-155 levels in serum exosomes may be used to diagnose epilepsy in early stages to assess epilepsy severity (Liu et al., 2022). Levetiracetam also inhibits M2 polarization of microglia by blocking abnormal neuronal activation and reducing miR-200c-3p levels in exosomes, which inhibits the protumorigenic effects of M2 microglia (Guo et al., 2024). Exosomes, as natural therapeutic vectors with an excellent ability to cross the BBB, can be used to efficiently deliver therapeutic miRNAs. miR-142-3p, miR-223-3p and miR-21-5p levels were found to be significantly increased in epileptogenic TSC lesions and to contain nucleic acid motifs that activate toll-like receptors (TLR7/8), allowing them to activate a neuroinflammatory response. These results provide new evidence for the role of exosomes and noncoding RNA cargo in the neuroinflammatory cascade in epilepsy and may help advance the development of novel biomarkers and therapeutics for refractory epilepsy (Cukovic et al., 2021). Liu et al. (2024) demonstrated that exosomes loaded with miR-129-5p significantly attenuate neurodegeneration in a mouse model of persistent epilepsy and reduce neuronal damage in the CA3 region in the epileptic brain by inhibiting HMGB1/TLR4-mediated neuroinflammation. miR-23b-3p interacts with the 3’-UTRs of STAT1 and GlyR1 to inhibit inflammatory factor expression in M1 microglia, and miR-23b-3p -loaded exosomes derived from adipose-derived stem cells (ADSCs) alleviate KA-induced inflammation in mice with epilepsy (Yang et al., 2024). Although exosomal miRNAs show great potential in treating epilepsy, there are still many related challenges, including the need to standardize of exosome sources, improve target delivery efficiency, and assess long-term safety. Future studies involving the use of multiomics techniques to screen specific miRNAs and optimize exosome engineering strategies are needed to facilitate clinical translation.

## 6 Future directions in epilepsy research: decoding glial RNA networks for therapeutic innovation

Although important progress has been made in understanding the mechanisms by which miRNAs and lncRNAs regulate glial cell function and neuroinflammation in epilepsy, the clinical translation of related therapies still faces several challenges. First, current evidence on glial ncRNA mechanisms relies predominantly on mouse models, limiting the translatability of glial findings to humans. Expanding investigations to diverse animal systems is imperative to validate pathophysiological relevance. Second, miRNAs and lncRNAs act through a complex multi-target network, and the inhibition of glial inflammation may also inhibit the phagocytosis of glial cells, which is not conducive to the control of epilepsy. Therefore, the mechanism of action of these molecules should be further explored (Huang et al., 2015). Third, although exosomes can penetrate the BBB, they have low yields and unstable drug delivery efficiencies, and free miRNAs are easily degraded in body fluids. The use of exosomes for therapeutic delivery remains a bottleneck (Wortzel et al., 2019; Liu et al., 2024).
